# Economic evaluations of neoadjuvant drug regimens in cancer: a systematic review

**DOI:** 10.3389/fpubh.2026.1918274

**Published:** 2026-08-19

**Authors:** Ying Nie, Xiaohui Wang, Shuo Kang, Zhenhua Pan

**Affiliations:** 1 School of Pharmacy, Hebei Medical University, Shijiazhuang, Hebei, China; 2 Pharmaceutical Management Research Center of Hebei Medical University, Shijiazhuang, Hebei, China; 3 Medical Insurance Office, The Second Hospital of Hebei Medical University, Shijiazhuang, Hebei, China; 4 School of Basic Medical Sciences, Hebei Medical University, Shijiazhuang, Hebei, China

**Keywords:** cancer, cost-effectiveness analysis, drug regimen, economic evaluation, neoadjuvant therapy, systematic review

## Abstract

**Background:**

Neoadjuvant drug therapy has become an important component of multidisciplinary cancer treatment, but newer targeted, immunotherapy-based, and multi-agent regimens are often associated with higher costs and uncertainty regarding long-term economic value. This systematic review evaluates the economic evidence for neoadjuvant drug regimens in cancer and summarizes key cost-effectiveness findings across tumor types and healthcare settings.

**Methods:**

We systematically searched PubMed, Embase, and the Cochrane Central Register of Controlled Trials (CENTRAL) from database inception to June 1, 2026. Eligible studies were full economic evaluations comparing neoadjuvant drug regimens or assessing the addition of a specific drug component to a baseline neoadjuvant regimen in patients with cancer. Studies comparing neoadjuvant drug regimens with non-drug-based or non-neoadjuvant strategies were excluded. Study selection and data extraction were performed independently by two reviewers. Reporting completeness was assessed using the CHEERS 2022 checklist, potential sources of bias were assessed using the ECOBIAS checklist, and findings were narratively synthesized.

**Results:**

A total of 2,572 records were identified, and 7 studies met the inclusion criteria. The included studies were published between 2015 and 2024 and involved breast cancer, muscle-invasive bladder cancer, and borderline resectable or locally advanced pancreatic cancer. Five studies focused on breast cancer, mainly HER2-positive disease. Most studies used model-based approaches and quality-adjusted life-years as the primary outcome. Pertuzumab-based neoadjuvant regimens for HER2-positive breast cancer and FOLFIRINOX for pancreatic cancer were generally reported to be cost-effective in their respective healthcare settings. In contrast, neoadjuvant pembrolizumab for muscle-invasive bladder cancer and AC-TH compared with TCH for breast cancer were not cost-effective under base-case assumptions. Key drivers included pathological complete response, recurrence or progression risk, drug costs, utility values, adverse event costs, time horizon, and assumptions linking short-term efficacy to long-term outcomes.

**Conclusion:**

Current economic evidence remains limited and concentrated mainly in HER2-positive breast cancer. Cost-effectiveness conclusions depend strongly on drug prices, downstream treatment costs, and model-based extrapolation of early efficacy endpoints. Future studies should incorporate long-term follow-up and real-world evidence.

**Systematic review registration:**

https://www.crd.york.ac.uk/PROSPERO/view/CRD420261462564, Identifier CRD420261462564.

## Introduction

Malignant tumors are among the leading public health challenges worldwide and remain a major cause of mortality and disease burden (1). According to GLOBOCAN 2022 (2), there were nearly 20 million new cancer cases and 9.7 million cancer deaths globally in 2022, and the cancer burden is expected to continue increasing as a result of population aging, population growth, and ongoing exposure to risk factors (3). Although advances in cancer diagnosis and treatment, including the combined use of surgery, radiotherapy, and systemic therapy, have improved outcomes for some patients, important challenges remain for patients with locally advanced solid tumors or tumors at high risk of recurrence. In these populations, local treatment alone or postoperative adjuvant therapy may be insufficient to prevent recurrence and metastasis or to achieve durable survival benefits.

Neoadjuvant therapy refers to systemic treatment delivered before definitive local therapy, particularly before surgery. It has become an important component of multidisciplinary care for a range of solid tumors. The main objectives of neoadjuvant therapy are to reduce tumor burden before surgery, improve resectability and the likelihood of R0 resection, and assess treatment response using indicators such as pathological complete response and tumor downstaging (4). Compared with postoperative adjuvant therapy alone, neoadjuvant therapy may also provide earlier control of occult micrometastatic disease, enable *in vivo* assessment of drug sensitivity, and inform subsequent individualized treatment decisions. Its clinical value has therefore attracted considerable attention in breast cancer, bladder cancer, pancreatic cancer, and other solid tumors (5).

With rapid progress in anticancer drug development (6), neoadjuvant treatment has expanded beyond conventional cytotoxic chemotherapy to include targeted therapies, immune checkpoint inhibitor-based combinations, and multi-agent regimens (7). These newer drug strategies offer opportunities to improve tumor response, surgical outcomes, and long-term survival (8, 9). At the same time, they increase drug acquisition costs, treatment management costs, and costs associated with adverse event management. In resource-constrained health systems, balancing clinical benefit against economic burden has become an unavoidable issue in the selection of neoadjuvant treatment strategies (10).

Pharmacoeconomic evaluation is a research approach that compares treatment costs with health outcomes, commonly using metrics such as quality-adjusted life-years and incremental cost-effectiveness ratios to assess the economic value of alternative interventions (11). In the neoadjuvant setting, economic evaluation can quantify not only the costs and short-term efficacy of drug regimens but also, through model-based extrapolation, their longer-term effects on recurrence, metastasis, survival, and quality of life (12). Pharmacoeconomic evidence can therefore support value assessment, clinical pathway optimization, and the allocation of health resources across competing neoadjuvant treatment options.

Although pharmacoeconomic studies in oncology have increased in recent years, existing reviews have paid relatively limited attention to neoadjuvant treatment strategies, particularly neoadjuvant drug regimens themselves (13). The available studies are dispersed across different tumor types, drug classes, and healthcare financing systems, and they vary in design, model structure, time horizon, cost components, and outcome measures (14). These differences make evidence synthesis and cross-study comparison challenging. A systematic review of economic evaluations of neoadjuvant drug strategies is therefore needed to characterize the existing evidence, summarize cost-effectiveness conclusions and key drivers, and provide more systematic evidence for clinical decision-making and health policy development.

## Methods

### Search strategy

This systematic review was conducted and reported in accordance with the PRISMA (Preferred Reporting Items for Systematic Reviews and Meta-Analyses) guidelines (15). PubMed, Embase, and the Cochrane Central Register of Controlled Trials (CENTRAL) were searched from database inception to June 1, 2026. The search strategies combined controlled vocabulary terms with free-text terms related to neoadjuvant or preoperative treatment, economic evaluation, and cancer. No publication-date or language restrictions were applied at the database-search stage. The complete electronic search strategies for all databases are provided in Supplementary Table S1.

The reference lists of the included studies and relevant reviews were examined manually to identify additional eligible publications. No separate search of grey-literature databases or other unpublished sources was undertaken. All retrieved records were imported into EndNote 21 for reference management and deduplication. The review was retrospectively registered in PROSPERO (CRD420261462564). No separate review protocol was published.

### Inclusion and exclusion criteria

Eligibility criteria were developed according to the PICO (Patient/Population, Intervention, Comparison, and Outcomes) framework. The population comprised patients with cancer receiving neoadjuvant drug therapy. Eligible studies were those evaluating the economic value of different neoadjuvant drug regimens or the addition of a specific drug component to a baseline neoadjuvant drug regimen. Eligible study designs included cost-effectiveness analyses, cost-utility analyses, cost-minimization analyses, and other full economic evaluations reporting incremental cost-effectiveness results. Only full-text articles published in English were eligible for inclusion.

Studies were excluded if they were not economic evaluations; did not include patients with cancer; were not conducted in the neoadjuvant drug treatment setting; compared neoadjuvant therapy with upfront surgery, adjuvant therapy, perioperative therapy, induction therapy, watch-and-wait strategies, or radiotherapy/chemoradiotherapy-based strategies rather than comparing neoadjuvant drug regimens; were cost-consequence analyses without reporting incremental cost-effectiveness ratios; were reviews, editorials, letters, conference abstracts, protocols, trial registrations, duplicate publications, or studies without accessible full text. When multiple publications were based on the same economic model or research question, the most complete report was retained.

### Study selection and data extraction

Study selection was performed independently by two reviewers. Titles were first screened, followed by abstract screening and full-text review of potentially eligible articles. To improve rigor and minimize errors, data from all included studies were extracted independently by two reviewers. Discrepancies were resolved through discussion, and a third senior reviewer was consulted when consensus could not be reached. The study selection process is presented in the PRISMA flow diagram.

Data extraction focused on key methodological characteristics, including study perspective, time horizon, cost type, treatment approach, and discount rate. Information on model type, cycle length, health outcome, willingness-to-pay threshold, incremental cost-effectiveness ratio, base-case result, and sensitivity analysis was also extracted.

### Assessment of reporting completeness and potential bias

The reporting completeness of the included studies was assessed using the CHEERS 2022 checklist (16). This checklist includes 28 items covering the title, abstract, introduction, methods, results, discussion, and other relevant information. For qualitative synthesis, studies that adequately addressed 25 or more of the 28 items were considered to have good reporting quality.

Potential sources of bias in the included economic evaluations were assessed using the ECOBIAS checklist (17). Each item was rated as “Yes,” “Partly,” “No,” “Unclear,” or “Not applicable.” A “Yes” response indicated that the issue specified in the corresponding question was adequately addressed and did not indicate that the named bias was present. No aggregate score or overall risk-of-bias category was assigned.

### Data analysis

Given the substantial heterogeneity across included studies in model structure, methodological approach, and outcome reporting, quantitative synthesis was considered inappropriate. A narrative synthesis was therefore conducted. Results were summarized in tabular form by systematically comparing key study characteristics, including model type, cost input, perspective, time horizon, discount rate, and main conclusions. The CHEERS 2022 and ECOBIAS assessments were summarized descriptively.

## Results

### Overview of included studies

**Figure 1:**
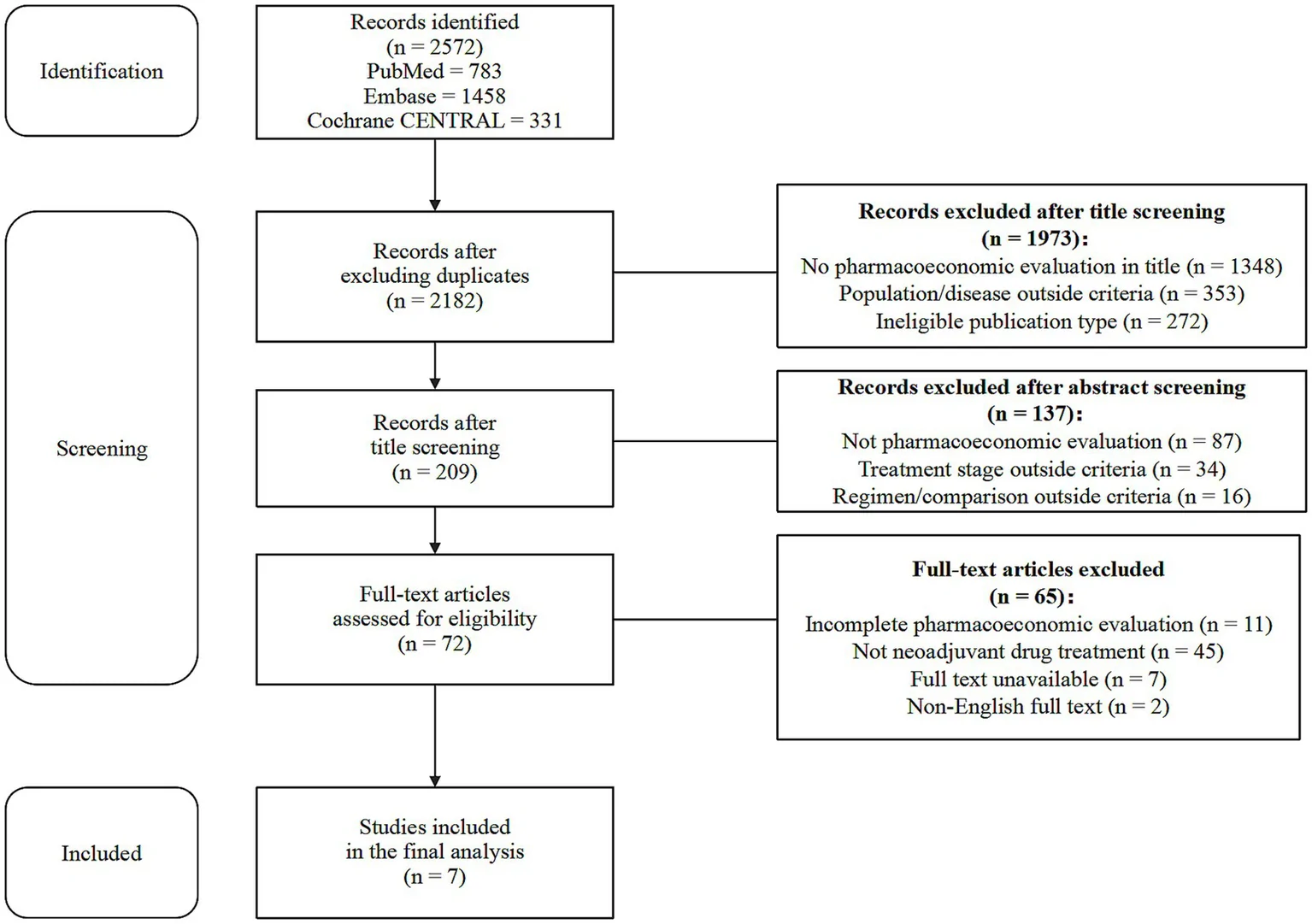
Flowchart of the study selection process.

The database search identified 2,572 records, including 783 from PubMed, 1,458 from Embase, and 331 from the Cochrane Library. After deduplication in EndNote 21, 2,182 records remained. Title screening excluded 1,973 records because the title did not indicate a pharmacoeconomic evaluation (*n* = 1,348), the population or disease was outside the eligibility criteria (*n* = 353), or the publication type was ineligible (*n* = 272), leaving 209 records for further screening. A further 137 records were excluded after abstract screening, and 72 full-text articles were assessed for eligibility. At the full-text stage, 65 articles were excluded: 11 were not full pharmacoeconomic evaluations, 45 were not related to neoadjuvant treatment, 7 full texts could not be obtained, and 2 were non-English publications. Ultimately, 7 studies were included in the qualitative synthesis (18–24). The study selection process is shown in Figure 1.

### Basic characteristics, reporting completeness, and risk-of-bias assessment of included studies

The included studies were published between 2015 and 2024. Two studies were published in 2015 (19, 23), one in 2020 (24), two in 2021 (20, 22), one in 2022 (21), and one in 2024 (18). All 7 studies were published in English and were conducted in Singapore (18), Canada (19), the United States (20, 21, 24), China (22), and Iran (23). Reporting completeness was assessed using the CHEERS 2022 checklist. Two studies had relatively high reporting completeness, whereas the remaining five studies had moderate reporting completeness. Detailed item-level results are provided in Supplementary Table S2.

The ECOBIAS assessment identified potential concerns related mainly to limited time horizons, the identification and incorporation of model inputs, treatment-effect estimates, assessment of uncertainty, and internal model consistency. Several items were rated as unclear because of insufficient reporting. Detailed item-level results are provided in Supplementary Table S3.

Most studies adequately reported the study objective, intervention comparison, model structure, perspective, time horizon, costs and health outcomes, incremental cost-effectiveness ratio, and sensitivity analyses. Frequently underreported items included heterogeneity, distributional effects, stakeholder engagement, and, in some studies, discount rates and cost years. The key methodological and structural characteristics of the included studies are summarized in Table 1.

**Table 1:** Characteristics of the included studies.

Study (author, year)	Cancer type/population	Intervention	Comparator	Model	Perspective	Time horizon	Cycle length	Cost input	Health outcome	WTP threshold	ICER	Base-case conclusion	Sensitivity analysis
Lim et al., 2024 (18)	HER2-positive early breast cancer	Pertuzumab + trastuzumab biosimilar + chemotherapy	Trastuzumab biosimilar + chemotherapy	Six-state Markov model	Singapore healthcare system	Lifetime	1 month	Direct medical costs	QALY	S$45,000/QALY	S$5,121/QALY	Cost-effective	OWSA, PSA, SA
Attard et al., 2015 (19)	Locally advanced, inflammatory, or early HER2-positive breast cancer	Pertuzumab + trastuzumab + chemotherapy	Trastuzumab + chemotherapy	Markov model	Canadian healthcare payer	28 years	1 month	Direct medical costs	QALY	CAD $100,000/QALY	CAD $25,388–46,196/QALY	Cost-effective	DSA, PSA, SA
Khaki et al., 2021 (20)	Muscle-invasive bladder cancer	Pembrolizumab	ddMVAC	Decision-tree model	US healthcare payer/third-party payer	2 years	NA	Direct drug costs	2-year RFS	$100,000/2-year RFS	$522,143/2-year RFS	Not cost-effective	Threshold analysis, OWSA, PSA
Ingram et al., 2022 (21)	Borderline resectable or locally advanced pancreatic ductal adenocarcinoma	FOLFIRINOX	G-nP	Decision-analytic mathematical model	US healthcare system	12 years	1 month	Direct medical costs	QALY	$100,000/QALY	$60,856.47/QALY	Cost-effective	OWSA, PSA
Xu et al., 2021 (22)	HER2-overexpressing breast cancer	AC-TH	TCH	Markov model	Chinese payer	5 years	1 year	Direct medical costs	QALY	$20,000/QALY	$52,565/QALY	Not cost-effective	OWSA, PSA
Hatam et al., 2015 (23)	Breast cancer receiving neoadjuvant chemotherapy	AC	PG	Decision-tree model	Iranian community perspective	1 year	NA	Direct medical, direct non-medical, and indirect costs	QALY	NR	$5,535/QALY	AC was dominant over PG	OWSA, PSA
Hassett et al., 2020 (24)	Stage II-III HER2-positive breast cancer	TCHP; THP + AC; THP; TH; T-DM1 + P	Multiple neoadjuvant strategy comparisons	Two discrete-time Markov models	US Medicare payer	5 years	1 week	Direct medical costs	QALY	NR	>$18 million/QALY (THP vs. TH, HR-negative); TH dominated THP (HR-positive)	TH most cost-effective	SA

AC, doxorubicin plus cyclophosphamide; AC-TH, doxorubicin plus cyclophosphamide followed by paclitaxel plus trastuzumab; CAD, Canadian dollars; ddMVAC, dose-dense methotrexate, vinblastine, doxorubicin, and cisplatin; DSA, deterministic sensitivity analysis; FOLFIRINOX, leucovorin, fluorouracil, irinotecan, and oxaliplatin; G-nP, gemcitabine plus nab-paclitaxel; HER2, human epidermal growth factor receptor 2; HR, hormone receptor; ICER, incremental cost-effectiveness ratio; NA, not applicable; NR, not reported; OWSA, one-way sensitivity analysis; PG, gemcitabine plus paclitaxel; PSA, probabilistic sensitivity analysis; QALY, quality-adjusted life-year; RFS, recurrence-free survival; SA, scenario analysis; S$, Singapore dollars; TCH, docetaxel, carboplatin, and trastuzumab; TCHP, docetaxel, carboplatin, trastuzumab, and pertuzumab; T-DM1 + P, trastuzumab emtansine plus pertuzumab; TH, paclitaxel plus trastuzumab; THP, paclitaxel, trastuzumab, and pertuzumab; THP+AC, paclitaxel, trastuzumab, and pertuzumab followed by doxorubicin plus cyclophosphamide; WTP, willingness-to-pay.

### Economic evaluation results

All included studies evaluated neoadjuvant drug regimens in cancer, although they differed in tumor type, intervention type, and health outcome (18–24). Five of the 7 studies focused on neoadjuvant treatment for breast cancer, including anti-HER2 therapy for HER2-positive breast cancer and comparisons of conventional chemotherapy regimens (18, 19, 22–24). The remaining 2 studies evaluated neoadjuvant immunotherapy for muscle-invasive bladder cancer (20) and neoadjuvant chemotherapy for borderline resectable or locally advanced pancreatic cancer (21). In terms of intervention type, the studies included both add-on evaluations of targeted drugs to baseline neoadjuvant regimens (18, 19) and head-to-head comparisons of different neoadjuvant chemotherapy regimens or treatment-intensity strategies (21–24). Except for the study by Khaki et al., which used 2-year recurrence-free survival as the main health outcome (20), all studies used quality-adjusted life-years as the principal measure of health benefit (18, 19, 21–24).

Overall, most studies suggested that selected neoadjuvant drug regimens may be cost-effective, but economic conclusions were not determined solely by drug class or treatment intensity. Instead, they were jointly influenced by incremental efficacy, drug prices, downstream costs of recurrence or metastatic treatment, and model-based extrapolation. In HER2-positive breast cancer, studies evaluating the addition of pertuzumab generally supported its economic value (18, 19). These models linked improved pathological complete response in the neoadjuvant phase to lower subsequent risks of recurrence or metastasis, and then estimated the resulting gains in QALYs and savings in downstream treatment costs. By contrast, Hassett et al. suggested that, when postoperative treatment can be adapted according to pathological response, increasing the intensity of neoadjuvant therapy does not necessarily improve economic value; de-escalated strategies may achieve similar health benefits at lower cost (24).

Findings for conventional chemotherapy regimens were more heterogeneous. Hatam et al. found that AC was less costly and more effective than PG, making AC a dominant strategy (23). Xu et al. compared AC-TH with TCH and highlighted the trade-off between efficacy, toxicity, and cost; interpretation of economic value depended on the model assumptions and willingness-to-pay threshold (22). Among non-breast cancer studies, Ingram et al. found that neoadjuvant FOLFIRINOX was more cost-effective than gemcitabine plus nab-paclitaxel, mainly because of improved survival, progression-free survival, and resection-related outcomes (21). In contrast, Khaki et al. reported that pembrolizumab provided only limited incremental gain in 2-year recurrence-free survival compared with ddMVAC while incurring substantially higher drug costs, and therefore did not show a cost-effectiveness advantage (20).

Taken together, the included studies indicate that the economic value of neoadjuvant drug therapy is closely related to early efficacy improvements, extrapolated long-term clinical benefits, and downstream changes in treatment costs. Because the evidence was concentrated in a limited number of tumor types and most studies relied on model-based extrapolation to estimate long-term outcomes, ICERs and cost-effectiveness conclusions should not be directly compared across studies without considering tumor characteristics, treatment pathways, study perspective, and model assumptions.

### Sensitivity analyses

The included studies generally used sensitivity analyses to assess parameter uncertainty (18–24). Most studies conducted one-way or deterministic sensitivity analyses and probabilistic sensitivity analyses (18–23), and some further performed scenario or threshold analyses (18–21, 24). Key drivers of the economic conclusions included pathological complete response rate, risk of recurrence or progression, drug cost, health-state utility values, adverse event management costs, and time horizon.

In HER2-positive breast cancer, pathological complete response and its association with long-term outcomes were among the most important sources of uncertainty (18, 19, 24). The economic value of pertuzumab-based regimens largely depended on whether improvements in neoadjuvant pathological complete response translated into lower recurrence rates and long-term QALY gains. For high-cost regimens with limited incremental efficacy, drug price had a particularly strong influence on conclusions. The threshold analysis by Khaki et al. showed that pembrolizumab would require a substantial price reduction to meet the prespecified economic threshold, indicating considerable uncertainty regarding the economic value of immunotherapy in the neoadjuvant setting (20).

Other influential factors included recurrence rates, metastatic transition probabilities, treatment-related adverse event costs, and surgery-related costs. The pancreatic cancer study showed that the cost-effectiveness of FOLFIRINOX was sensitive to treatment-related adverse event costs and recurrence-related parameters (21). Studies of conventional chemotherapy regimens also indicated that utility values and cost parameters could affect model results (22, 23). Overall, sensitivity analyses suggested that neoadjuvant economic evaluations should not focus only on drug prices; early efficacy endpoints, long-term outcome extrapolation, subsequent treatment pathways, and cost data sources are also central determinants of model stability.

## Discussion

This study systematically reviewed economic evaluations of neoadjuvant drug regimens in cancer. The available evidence remains limited and is concentrated mainly in HER2-positive breast cancer, with a small number of studies in muscle-invasive bladder cancer and borderline resectable or locally advanced pancreatic cancer. Most studies used model-based approaches to estimate the long-term economic value of neoadjuvant drug regimens and reported QALYs and ICERs as key outcomes (25). Overall, selected neoadjuvant targeted therapies or chemotherapy regimens were cost-effective within specific national healthcare systems and willingness-to-pay thresholds. However, studies differed substantially in tumor type, intervention, perspective, cost scope, time horizon, and model structure, and their findings should therefore not be compared directly without careful contextual interpretation.

Our review suggests that the economic value of neoadjuvant drug therapy is not determined solely by the acquisition cost of the drug. Rather, it depends on whether higher upfront treatment costs can be offset by long-term clinical benefits and downstream cost savings. In HER2-positive breast cancer, studies of add-on pertuzumab generally reported favorable economic results, mainly because higher pathological complete response rates were linked to lower subsequent risks of recurrence or metastasis and long-term QALY gains. In contrast, in the study of neoadjuvant immunotherapy for muscle-invasive bladder cancer, pembrolizumab generated only a limited incremental improvement in 2-year recurrence-free survival over platinum-based chemotherapy while substantially increasing drug costs, and was therefore not cost-effective. These findings highlight that, in the neoadjuvant setting, the translation of short-term efficacy gains into long-term benefit is a key determinant of economic value (26).

From a methodological perspective, the included studies commonly relied on model-based extrapolation to translate short- or intermediate-term outcomes, such as pathological complete response, recurrence-free survival, and R0 resection rate, into long-term survival, QALYs, and costs. This approach is often necessary in neoadjuvant economic evaluations because mature long-term survival data are frequently unavailable at the time of assessment (27). Nevertheless, it introduces substantial uncertainty. The strength of the association between pathological complete response and long-term recurrence, metastasis, or survival may vary by tumor type, molecular subtype, and treatment regimen. Overreliance on surrogate endpoints may therefore overestimate or underestimate long-term benefit. Future studies should more clearly justify the evidence linking early efficacy endpoints to long-term outcomes and should test the robustness of model assumptions using scenario analyses, threshold analyses, and real-world data (28). The ECOBIAS assessment further indicated that the interpretation of the included studies may be affected by limitations in time horizon, treatment-effect evidence, model-input identification, uncertainty assessment, and internal consistency. In addition, several items could not be clearly assessed because relevant information was insufficiently reported. These findings should be interpreted as potential sources of bias or uncertainty rather than as an overall judgment of study quality.

Variation in cost components was another important factor limiting comparability across studies. Some studies included only drug costs or direct medical costs, whereas others also incorporated adverse event management, treatment after recurrence or metastasis, end-of-life care, direct non-medical costs, and indirect costs. Differences across countries and regions in drug prices, reimbursement policies, healthcare service costs, and treatment pathways mean that the same neoadjuvant regimen may yield different economic conclusions in different healthcare systems. Accordingly, ICERs derived from one country or payer setting should not be directly generalized to other settings. In the Chinese context, centralized drug procurement, national reimbursement negotiations, domestic biosimilars, and access to subsequent therapies may substantially alter the cost-effectiveness of neoadjuvant drug regimens (29).

This review also showed that the current evidence is unevenly distributed across tumor types and drug classes. The included studies were concentrated in breast cancer, especially HER2-positive breast cancer, whereas economic evaluations of rapidly expanding neoadjuvant strategies such as immunotherapy, immunochemotherapy, and targeted combination therapy remain relatively limited in lung cancer, gastric cancer, esophageal cancer, melanoma, and other solid tumors. As neoadjuvant treatment continues to expand across malignancies, economic evidence derived from adjuvant or advanced disease settings alone is unlikely to meet clinical and reimbursement decision-making needs. Future studies should incorporate the latest clinical trial results and real-world evidence and should address specific tumor types, biomarker-defined populations, and treatment sequences.

This study has several limitations. First, although multiple databases were searched without language restrictions and reference lists were manually screened, only English-language full-text articles were eligible for inclusion. Unpublished studies and conference abstracts were not included, and some relevant studies may therefore have been missed. Second, the number of included studies was small, and substantial heterogeneity in tumor type, treatment regimen, model structure, and outcome measure precluded quantitative synthesis. Third, this review was based on published studies and used narrative synthesis; model parameters were not recalculated or standardized. Differences in cost year, currency, willingness-to-pay threshold, and cost scope may therefore affect cross-study comparisons. Finally, some included studies relied on clinical trial data or literature-based parameters for long-term extrapolation, and the effects of real-world treatment pathways, adherence, drug price changes, and subsequent treatment patterns require further validation. In addition, the CHEERS 2022 categories were defined for descriptive purposes in this review and should not be interpreted as an official quality score. Some ECOBIAS items could not be clearly assessed because of insufficient reporting in the original studies.

In light of these limitations, future economic evaluations of neoadjuvant drug therapy should improve methodological consistency and decision relevance. Long-term follow-up data and real-world evidence should be used to validate the relationships between early endpoints, such as pathological complete response, recurrence-free survival, and R0 resection, and long-term survival, quality of life, and subsequent treatment costs. Studies should also fully report model structure, parameter sources, cost year, discount rate, willingness-to-pay threshold, and sensitivity analyses to improve transparency and comparability. As neoadjuvant immunotherapy, targeted therapy, and combination regimens expand across solid tumors, more targeted economic evaluations are needed across tumor types, biomarker-defined populations, and healthcare financing systems.

## Conclusion

Economic evaluations of neoadjuvant drug regimens in cancer remain limited, and the existing evidence is concentrated mainly in HER2-positive breast cancer, with fewer studies in muscle-invasive bladder cancer and pancreatic cancer. The included studies suggest that selected neoadjuvant targeted therapies or chemotherapy regimens may be cost-effective within specific healthcare systems and willingness-to-pay thresholds. However, their economic value depends strongly on the translation of early efficacy endpoints into long-term clinical benefit, drug prices, downstream treatment costs, and model extrapolation assumptions. Potential limitations in model inputs, treatment-effect evidence, and uncertainty assessment should also be considered when interpreting these findings. Given the heterogeneity in tumor type, intervention, cost scope, time horizon, and model structure, current findings should not be compared directly across studies. Future research should incorporate long-term follow-up data and real-world evidence to evaluate neoadjuvant drug regimens across tumor types, treatment strategies, and healthcare systems, thereby providing more robust evidence for treatment selection and health resource allocation.

## Data Availability

The original contributions presented in the study are included in the article/Supplementary material, further inquiries can be directed to the corresponding author/s.
