# Supplementary material for: Economic evaluations of neoadjuvant drug regimens in cancer: a systematic review
Source: Front Public Health. 2026 Aug 19;14:1918274. doi: 10.3389/fpubh.2026.1918274 (PMC13534116; doi:10.3389/fpubh.2026.1918274)
Supplement: Supplementary file 1 [file Table_1.DOCX]

**Supplementary Table S1. Full electronic search strategies**

Databases were searched from inception to 1 June 2026. No publication-date or language filters were applied at the database-search stage. The PubMed and Embase strategies are displayed as separate conceptual blocks for readability; the Boolean logic is unchanged from the executed searches.

**PubMed**

| Set | Search strategy |
| --- | --- |
| #1 | ("Neoadjuvant Therapy"[Mesh] OR neoadjuvant*[tiab] OR "preoperative treatment"[tiab] OR "preoperative therapy"[tiab] OR "pre-operative treatment"[tiab] OR "pre-operative therapy"[tiab] OR "induction therapy"[tiab] OR "induction treatment"[tiab] OR "preoperative chemotherapy"[tiab] OR "preoperative immunotherapy"[tiab] OR "preoperative chemoradiotherapy"[tiab] OR "neoadjuvant chemotherapy"[tiab] OR "neoadjuvant immunotherapy"[tiab] OR "neoadjuvant chemoradiotherapy"[tiab]) |
| #2 | ("Cost-Benefit Analysis"[Mesh] OR "Costs and Cost Analysis"[Mesh] OR "Economics"[Mesh] OR "cost-effectiveness"[tiab] OR "cost effectiveness"[tiab] OR "cost-utility"[tiab] OR "cost utility"[tiab] OR "cost-benefit"[tiab] OR "cost benefit"[tiab] OR "cost-minimization"[tiab] OR "cost minimization"[tiab] OR "economic evaluation"[tiab] OR "economic analysis"[tiab] OR pharmacoeconomic*[tiab] OR "health economics"[tiab] OR ICER[tiab] OR QALY[tiab] OR QALYs[tiab] OR "quality adjusted life year"[tiab] OR "quality-adjusted life year"[tiab] OR "quality adjusted life years"[tiab] OR "quality-adjusted life years"[tiab]) |
| #3 | ("Neoplasms"[Mesh] OR cancer*[tiab] OR tumor*[tiab] OR tumour*[tiab] OR neoplasm*[tiab] OR carcinoma*[tiab] OR adenocarcinoma*[tiab] OR malignan*[tiab] OR oncology[tiab]) |
| #4 | #1 AND #2 AND #3 |

**Embase**

| Set | Search strategy |
| --- | --- |
| #1 | ('neoadjuvant therapy'/exp OR neoadjuvant*:ti,ab,kw OR 'preoperative treatment':ti,ab,kw OR 'preoperative therapy':ti,ab,kw OR 'pre-operative treatment':ti,ab,kw OR 'pre-operative therapy':ti,ab,kw OR 'preoperative chemotherapy':ti,ab,kw OR 'preoperative immunotherapy':ti,ab,kw OR 'preoperative chemoradiotherapy':ti,ab,kw OR 'neoadjuvant chemotherapy':ti,ab,kw OR 'neoadjuvant immunotherapy':ti,ab,kw OR 'neoadjuvant chemoradiotherapy':ti,ab,kw OR 'induction chemotherapy':ti,ab,kw OR 'induction immunotherapy':ti,ab,kw OR 'induction chemoradiotherapy':ti,ab,kw) |
| #2 | ('cost effectiveness analysis'/exp OR 'cost benefit analysis'/exp OR 'cost utility analysis'/exp OR 'cost minimization analysis'/exp OR 'economic evaluation'/exp OR 'cost-effectiveness':ti,ab,kw OR 'cost effectiveness':ti,ab,kw OR 'cost-utility':ti,ab,kw OR 'cost utility':ti,ab,kw OR 'cost-benefit':ti,ab,kw OR 'cost benefit':ti,ab,kw OR 'cost-minimization':ti,ab,kw OR 'cost minimization':ti,ab,kw OR 'economic evaluation':ti,ab,kw OR pharmacoeconomic*:ti,ab,kw OR 'pharmacoeconomic analysis':ti,ab,kw OR 'health economic evaluation':ti,ab,kw OR icer:ti,ab,kw OR qaly:ti,ab,kw OR qalys:ti,ab,kw OR 'quality adjusted life year':ti,ab,kw OR 'quality-adjusted life year':ti,ab,kw OR 'quality adjusted life years':ti,ab,kw OR 'quality-adjusted life years':ti,ab,kw) |
| #3 | ('neoplasm'/exp OR cancer*:ti,ab,kw OR tumor*:ti,ab,kw OR tumour*:ti,ab,kw OR neoplasm*:ti,ab,kw OR carcinoma*:ti,ab,kw OR adenocarcinoma*:ti,ab,kw OR malignan*:ti,ab,kw OR oncology:ti,ab,kw) |
| #4 | #1 AND #2 AND #3 |

**Cochrane Central Register of Controlled Trials (CENTRAL)**

| Set | Search strategy |
| --- | --- |
| #1 | MeSH descriptor: [Neoadjuvant Therapy] explode all trees |
| #2 | neoadjuvant*:ti,ab,kw OR preoperative*:ti,ab,kw OR perioperative*:ti,ab,kw OR induction therap*:ti,ab,kw |
| #3 | #1 OR #2 |
| #4 | MeSH descriptor: [Costs and Cost Analysis] explode all trees |
| #5 | MeSH descriptor: [Economics, Medical] explode all trees |
| #6 | MeSH descriptor: [Cost-Benefit Analysis] explode all trees |
| #7 | "cost-effectiveness":ti,ab,kw OR "cost effectiveness":ti,ab,kw OR cost-utility:ti,ab,kw OR cost-benefit:ti,ab,kw OR cost-minimization:ti,ab,kw OR pharmacoeconomic*:ti,ab,kw OR economic evaluation*:ti,ab,kw OR health economic*:ti,ab,kw OR cost analys*:ti,ab,kw |
| #8 | #4 OR #5 OR #6 OR #7 |
| #9 | #3 AND #8 |

Abbreviations: CENTRAL, Cochrane Central Register of Controlled Trials; ICER, incremental cost-effectiveness ratio; QALY, quality-adjusted life-year.
