# Supplementary material for: Economic evaluations of neoadjuvant drug regimens in cancer: a systematic review
Source: Front Public Health. 2026 Aug 19;14:1918274. doi: 10.3389/fpubh.2026.1918274 (PMC13534116; doi:10.3389/fpubh.2026.1918274)
Supplement: Supplementary file 2 [file Table_2.DOCX]

**Supplementary Table S2. Item-level assessment of reporting completeness using the CHEERS 2022 checklist**

Each CHEERS 2022 item was coded as Yes, No, or not applicable (NA). Yes indicates that the item was addressed in the publication, either fully or partially; No indicates that no relevant information was reported. The binary coding and the descriptive thresholds below were operationally defined for this review and are not part of the official CHEERS 2022 guidance.

| **Item** | **CHEERS 2022 reporting item** | **Lim et al. (2024)** | **Attard et al. (2015)** | **Khaki et al. (2021)** | **Ingram et al. (2022)** | **Xu et al. (2021)** | **Hatam et al. (2015)** | **Hassett et al. (2020)** |
| --- | --- | --- | --- | --- | --- | --- | --- | --- |
| 1 | Title | Yes | Yes | Yes | Yes | Yes | Yes | Yes |
| 2 | Abstract | Yes | Yes | Yes | Yes | Yes | Yes | Yes |
| 3 | Background and objectives | Yes | Yes | Yes | Yes | Yes | Yes | Yes |
| 4 | Health economic analysis plan | Yes | No | No | No | No | No | No |
| 5 | Study population | Yes | Yes | Yes | Yes | Yes | Yes | Yes |
| 6 | Setting and location | Yes | Yes | Yes | Yes | Yes | Yes | Yes |
| 7 | Comparators | Yes | Yes | Yes | Yes | Yes | Yes | Yes |
| 8 | Perspective | Yes | Yes | Yes | Yes | Yes | Yes | Yes |
| 9 | Time horizon | Yes | Yes | Yes | Yes | Yes | Yes | Yes |
| 10 | Discount rate | Yes | Yes | Yes | Yes | Yes | Yes | No |
| 11 | Selection of outcomes | Yes | Yes | Yes | Yes | Yes | Yes | Yes |
| 12 | Measurement of outcomes | Yes | Yes | Yes | Yes | Yes | Yes | Yes |
| 13 | Valuation of outcomes | Yes | Yes | NA | Yes | Yes | Yes | Yes |
| 14 | Measurement and valuation of resources and costs | Yes | Yes | Yes | Yes | Yes | Yes | Yes |
| 15 | Currency, price date, and conversion | Yes | Yes | Yes | Yes | Yes | Yes | Yes |
| 16 | Rationale and description of model | Yes | Yes | Yes | Yes | Yes | Yes | Yes |
| 17 | Analytics and assumptions | Yes | Yes | Yes | Yes | Yes | Yes | Yes |
| 18 | Characterising heterogeneity | No | No | No | Yes | Yes | No | Yes |
| 19 | Characterising distributional effects | Yes | No | No | No | No | No | No |
| 20 | Characterising uncertainty | Yes | Yes | Yes | Yes | Yes | Yes | Yes |
| 21 | Approach to engagement with patients and others affected | Yes | Yes | No | No | No | No | No |
| 22 | Study parameters | Yes | Yes | Yes | Yes | Yes | Yes | Yes |
| 23 | Summary of main results | Yes | Yes | Yes | Yes | Yes | Yes | Yes |
| 24 | Effect of uncertainty | Yes | Yes | Yes | Yes | Yes | Yes | Yes |
| 25 | Effect of engagement | Yes | Yes | No | No | No | No | No |
| 26 | Study findings, limitations, generalisability, and current knowledge | Yes | Yes | Yes | Yes | Yes | Yes | Yes |
| 27 | Source of funding | Yes | Yes | Yes | Yes | Yes | No | Yes |
| 28 | Conflicts of interest | Yes | Yes | Yes | Yes | Yes | No | Yes |

Reporting completeness was classified as relatively high when 25–28 items were coded Yes, moderate when 20–24 items were coded Yes, and low when fewer than 20 items were coded Yes. These categories were used only for descriptive comparison and do not represent methodological quality or risk of bias. Abbreviations: CHEERS, Consolidated Health Economic Evaluation Reporting Standards; NA, not applicable.
