# Supplementary material for: Economic evaluations of neoadjuvant drug regimens in cancer: a systematic review
Source: Front Public Health. 2026 Aug 19;14:1918274. doi: 10.3389/fpubh.2026.1918274 (PMC13534116; doi:10.3389/fpubh.2026.1918274)
Supplement: Supplementary file 3 [file Table_3.DOCX]

**Supplementary Table S3. Item-level assessment of potential bias using the ECOBIAS checklist**

Each ECOBIAS question was rated as Yes, Partly, No, Unclear, or not applicable (NA). A Yes response indicates that the issue specified in the corresponding question was adequately addressed; it does not indicate that the named bias was present. No aggregate score or overall risk-of-bias category was assigned.

| **Item** | **ECOBIAS item** | **Lim et al. (2024)** | **Attard et al. (2015)** | **Khaki et al. (2021)** | **Ingram et al. (2022)** | **Xu et al. (2021)** | **Hatam et al. (2015)** | **Hassett et al. (2020)** |
| --- | --- | --- | --- | --- | --- | --- | --- | --- |
| A1 | Narrow perspective bias | Yes | Yes | Partly | Yes | Partly | Yes | Partly |
| A2 | Inefficient comparator bias | Yes | Yes | Yes | Yes | Yes | Partly | Yes |
| A3 | Cost measurement omission bias | Yes | Partly | No | Yes | Partly | Partly | Partly |
| A4 | Intermittent data collection bias | Yes | Yes | No | Yes | No | No | Partly |
| A5 | Invalid valuation bias | Partly | Partly | Yes | Yes | Partly | Partly | Partly |
| A6 | Ordinal ICER bias | Yes | Yes | Yes | Yes | Yes | Yes | Yes |
| A7 | Double-counting bias | Unclear | Unclear | Unclear | Unclear | Unclear | Unclear | Unclear |
| A8 | Inappropriate discounting bias | Yes | Yes | No | Yes | Yes | Yes | No |
| A9 | Limited sensitivity analysis bias | Partly | Partly | Partly | Partly | Partly | Partly | No |
| A10 | Sponsor bias | Partly | Partly | Partly | Partly | Partly | Partly | Partly |
| A11 | Reporting and dissemination bias | No | No | No | No | No | No | No |
| B1 | Structural assumptions bias | Yes | Partly | Partly | Yes | Partly | Partly | Partly |
| B2 | No-treatment comparator bias | Yes | Yes | Yes | Yes | Yes | Partly | Yes |
| B3 | Wrong model bias | Yes | Yes | Partly | Yes | Partly | Partly | Partly |
| B4 | Limited time horizon bias | Yes | Partly | No | Partly | No | No | No |
| B5 | Bias related to data identification | Partly | Partly | Partly | Partly | Partly | Partly | Partly |
| B6 | Bias related to baseline data | Partly | Partly | Partly | Yes | Partly | No | No |
| B7 | Bias related to treatment effects | Partly | Partly | No | Partly | Partly | No | No |
| B8 | Bias related to quality-of-life weights (utilities) | Partly | Partly | NA | Partly | Partly | No | No |
| B9 | Non-transparent data incorporation bias | Partly | Partly | Partly | Yes | Partly | No | Partly |
| B10 | Limited scope bias | Partly | Partly | Partly | Partly | Partly | No | No |
| B11 | Bias related to internal consistency | Unclear | Unclear | Unclear | Partly | Partly | Unclear | Partly |

Part A comprises general sources of bias in economic evaluations; Part B comprises model-specific sources of bias. Abbreviation: NA, not applicable.
